# Zonally asymmetric phytoplankton response to the Southern annular mode in the marginal sea of the Southern ocean

**DOI:** 10.1038/s41598-021-89720-4

**Published:** 2021-05-13

**Authors:** Kyung Min Noh, Hyung-Gyu Lim, Jong-Seong Kug

**Affiliations:** 1 Division of Environmental Science and Engineering, Pohang University of Science and Technology (POSTECH), 77 Cheongam-Ro Nam-Gu, Pohang, 790-784 South Korea; 2 Princeton University/Atmospheric and Oceanic Sciences Program, Princeton, NJ 08540 USA

**Keywords:** Climate sciences, Ocean sciences

## Abstract

Antarctic marine biological variability modulates climate systems via the biological pump. However, the knowledge of biological response in the Southern Ocean to climate variability still has been lack of understanding owing to limited ocean color data in the high latitude region. We investigated the surface chlorophyll concentration responses to the Southern annular mode (SAM) in the marginal sea of the Southern ocean using satellite observation and reanalysis data focusing on the austral summer. The positive phase of SAM is associated with enhanced and poleward-shifted westerly winds, leading to physical and biogeochemical responses over the Southern ocean. Our result indicates that chlorophyll has strong zonally asymmetric responses to SAM owing to different limiting factors of phytoplankton growth per region. For the positive SAM phase, chlorophyll tends to increase in the western Amundsen–Ross Sea but decreases in the D’Urville Sea. It is suggested that the distinct limiting factors are associated with the seasonal variability of sea ice and upwelling per region.

## Introduction

The Southern Annular Mode (SAM), also referred to as the Antarctic Oscillation, is the climate mode corresponding to the northern hemisphere’s Arctic Oscillation^1^. The SAM is defined as the first principal component of the empirical orthogonal function of the geopotential height or sea-level pressure anomalies^2^, so it is the most dominant climate mode of atmospheric circulation in the southern hemisphere^3, 4^ in addition to the ENSO^5^. The SAM represents the low-pressure cell positioned at the center of Antarctica and the high-pressure cell surrounding this low-pressure cell. The strong westerly wind blows along the strong gradient between the high- and low-pressure systems; therefore, the positive phase of SAM is associated with poleward-shifted and intensified jets^6^. The SAM not only dominates the southern hemisphere circulation but also contributes to air temperature warming and decreased precipitation at the 40°–60°S latitude and opposite signals with temperature and precipitation each north of 40°S^7, 8^.

The atmospheric circulation change due to SAM alters the Southern Ocean’s circulation patterns and biogeochemical cycling^9–13^. The intensified jet and its southward shift enhance the Ekman transport equatorward and the sea surface temperature (SST) decreases with stronger Ekman pumping above the 60°S region^8, 14^. Also, the mixed layer depth (MLD), net air-sea heat fluxes, sea ice, mesoscale eddies, and Atlantic meridional overturning circulation is strongly affected by SAM around the Southern Ocean^5, 13, 15–17^. These physical effects on the Southern Ocean could induce biogeochemical changes, such as changes in nutrient and phytoplankton concentration. However, thus far, our understanding of relationships between the biogeochemical and these climate variabilities in the Southern Ocean still remains insufficient.

Many studies about the interaction between climate variability and marine biological activity have been conducted mostly on tropical oceans^18–21^ and the Arctic Ocean^22–25^, but several researchers attempted to understand the Southern Ocean. Lovenduski and Gruber^14^ proposed the relationship of the Southern Ocean circulation and phytoplankton variabilities with SAM. They suggested that the biological responses associated with SAM are nearly zonally symmetric. Despite the nearly symmetric atmospheric forces in zonal direction, researchers recently argued the zonal asymmetric features and impacts of SAM on physical parameters, including MLD, wind, and temperature^26, 27^. In particular, Sallee^27^ demonstrated the different relationships between MLD and surface chlorophyll concentration in each ocean basin of the Subantarctic Zone, mentioning the inverse relationship between mixed layer depth and chlorophyll concentration.

Recently, manly researches about understanding phytoplankton phenology in the Southern Ocean have been conducted by analyzing various observations and model results^28–30^ focusing on the short time scale interaction between phytoplankton and physical and abiotic conditions of the ocean; light availability and nutrients abundance. In this study, we revisit biological responses depending on the SAM phase based on satellite observation and reanalysis data on an interannual timescale with a similar approach of physical and biogeochemical interactions in the Southern Ocean. These data are extended from 1998 to 2019 which are 10 years longer than that of Lovenduski and Gruber^14^. Owing to the extended period and seasonality, the physical responses to SAM are changed in the Southern Ocean that asymmetric phytoplankton response to the SAM became more distinct in the marginal sea of the Southern Ocean, which was weakly shown on the regression between chlorophyll and SAM in Lovenduski and Gruber^14^. We introduce the used observation and reanalysis data and the technique of treating each data. Subsequently, we suggest the zonally asymmetric chlorophyll response to SAM with detailed two different mechanisms through which SAM leads to inhomogeneous chlorophyll responses depending on each region. Finally, we discuss the implications of our results on the Southern Ocean.

## Methods

We used the satellite observation of chlorophyll $a$ concentration from the European Space Agency’s Ocean Colour Climate Change Initiative (ESA-CCI, version 3)^31^ from 1998 to 2019. To fairly compare the chlorophyll and other physical variables, the ESA-CCI chlorophyll data were re-gridded as a 1° grid using the first-order conservative remapping scheme using the Climate Data Operator (CDO) (Schulzweida et al., 2007). The chlorophyll distribution follows the natural log-normal distribution^32^ that the chlorophyll data were utilized by taking the natural logarithm after re-gridding, and means were calculated by the log-transformed data in this study. The other monthly mean climate data were obtained from different reanalysis data: the SST and sea ice concentration were obtained from the Hadley Centre’s Sea Ice and Sea Surface Temperature dataset (version 1.1)^33^. Moreover, the net surface shortwave flux at all sky was provided by the Clouds and the Earth’s Radiant Energy System (version 2.8)^34^.

The Ekman pumping velocity,$W_{E}$, is derived from the wind stress curl, where $\rho , f, {\text{and }}\tau$ represent the density, Coriolis coefficient, and wind stress, respectively.

$$W_{E} = \frac{1}{\rho } \nabla \times \left( {\frac{\tau }{f}} \right)$$

The wind stress data used for calculating the Ekman pumping velocity were obtained from the ERA-Interim data^35^. The MLD data were obtained from the Simple Ocean Data Assimilation ocean/sea-ice reanalysis (version 3) with density criteria (${\Delta }\sigma = 0.03 {\text{kg m}}^{ - 3}$)^36^. The SAM index is defined by Marshall^37^ (available at https://legacy.bas.ac.uk-6/met/gjma/sam.html), which is based on the meridional pressure difference between the latitudes of 40° S and 65° S study with station-observed data, was employed in this study.

All the reanalysis data were re-gridded using a bilinear interpolation method to a regular 1° $\times$ 1° grid. Because the satellite cannot obtain complete data during the autumn/winter season owing to the high solar zenith angle and high albedo by the sea ice and clouds, the averaged data of the austral summer season (Dec.–Feb.) were used for the analysis. To investigate the interannual relationship between chlorophyll and SAM, the climatological seasonal cycles and linear trends were all removed from the data at each grid cell.

## Results

**Figure 1:**
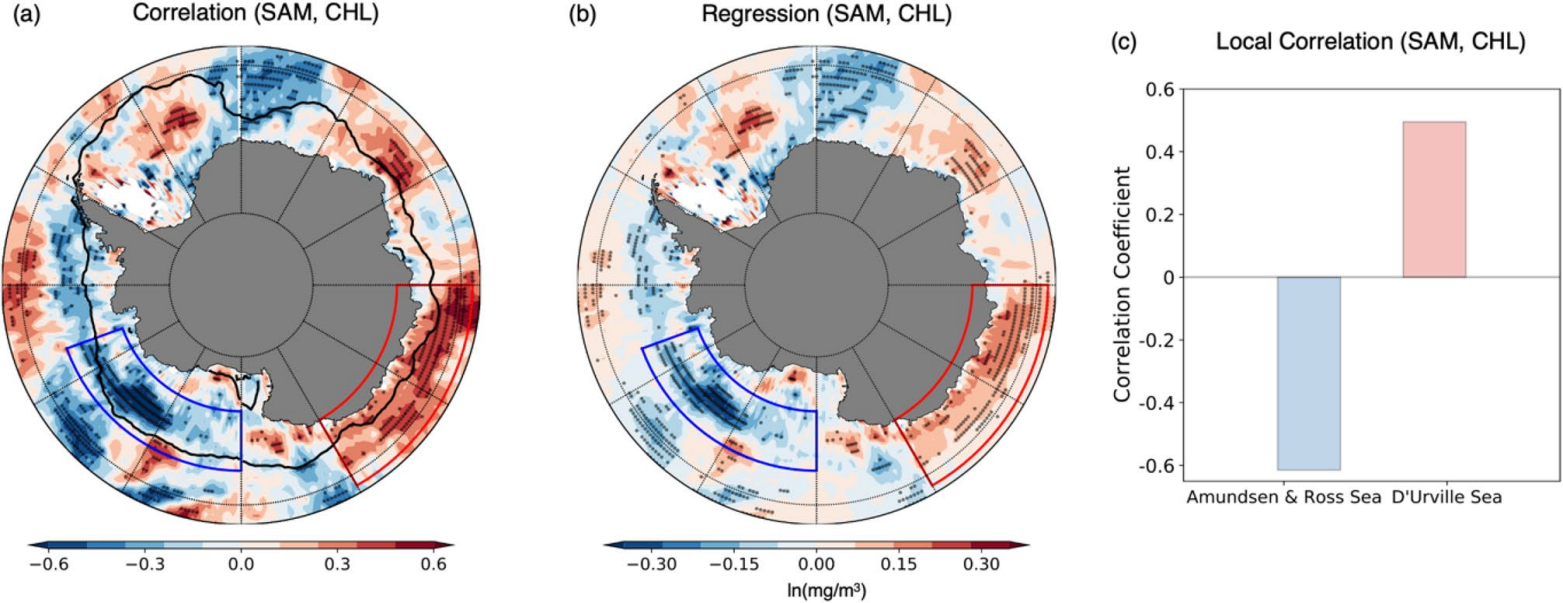
(**a**) Correlation and (**b**) regression map of the SAM index and chlorophyll concentration. The natural log of the chlorophyll concentration [${\text{ln}}\left( {{\text{mg}}/{\text{m}}^{ - 3} } \right)$] was used to estimate the correlation and regression. The thick solid line, which is the 15%-sea-ice concentration line, represents the climatology of sea ice extent in the austral summer for 1998–2019 period, and the 95% significant correlation (*p *value < 0.05) regions are expressed as black dots. White areas indicate the region where satellites have not observed chlorophyll for at least three years due to sea-ice cover or cloud. (**c**) The correlation coefficients between the SAM and natural log chlorophyll concentration that are spatially averaged over the western and eastern parts of the Southern Ocean are shown using blue and red boxes, respectively.

Figure 1 shows the zonally asymmetric response of the Southern Ocean phytoplankton to the SAM in the austral summer season over the Southern Ocean unlike nearly zonal-symmetric atmosphere circulation^8^, which is a distinctive feature of the Antarctic region. The positive phase of SAM is associated with the intensified and poleward-shifted westerly winds, leading to changes in the circulation and biological response of the Southern Ocean. Although the atmospheric circulation changes are almost homogeneous in the zonal direction, the biological responses are zonally asymmetric to a large extent. A significant negative correlation (Fig. 1a) and regression (Fig. 1b) exist in the western Antarctic Amundsen–Ross Sea region (110° W–180° W, 64° S–72° S), whereas a positive correlation and regression are significant in the eastern Antarctic D’Urville Sea region (120° E–180° E, 58° S–68° S). Further, an alternating pattern appears in part of the Southern Ocean between the South Atlantic and the Indian Ocean sectors (20° W–60° E, 60° S–67° S). The regions where strong signals are identified on a large scale are shown as the blue and red boxes showing correlation values of approximately − 0.61 and 0.50, respectively, exceeding the 95% confidence level (p-value < 0.05) (Fig. 1c). These regional asymmetric differences arise from the environmental differences of each part of the Southern Ocean, which are critical for phytoplankton growth.

**Figure 2:**
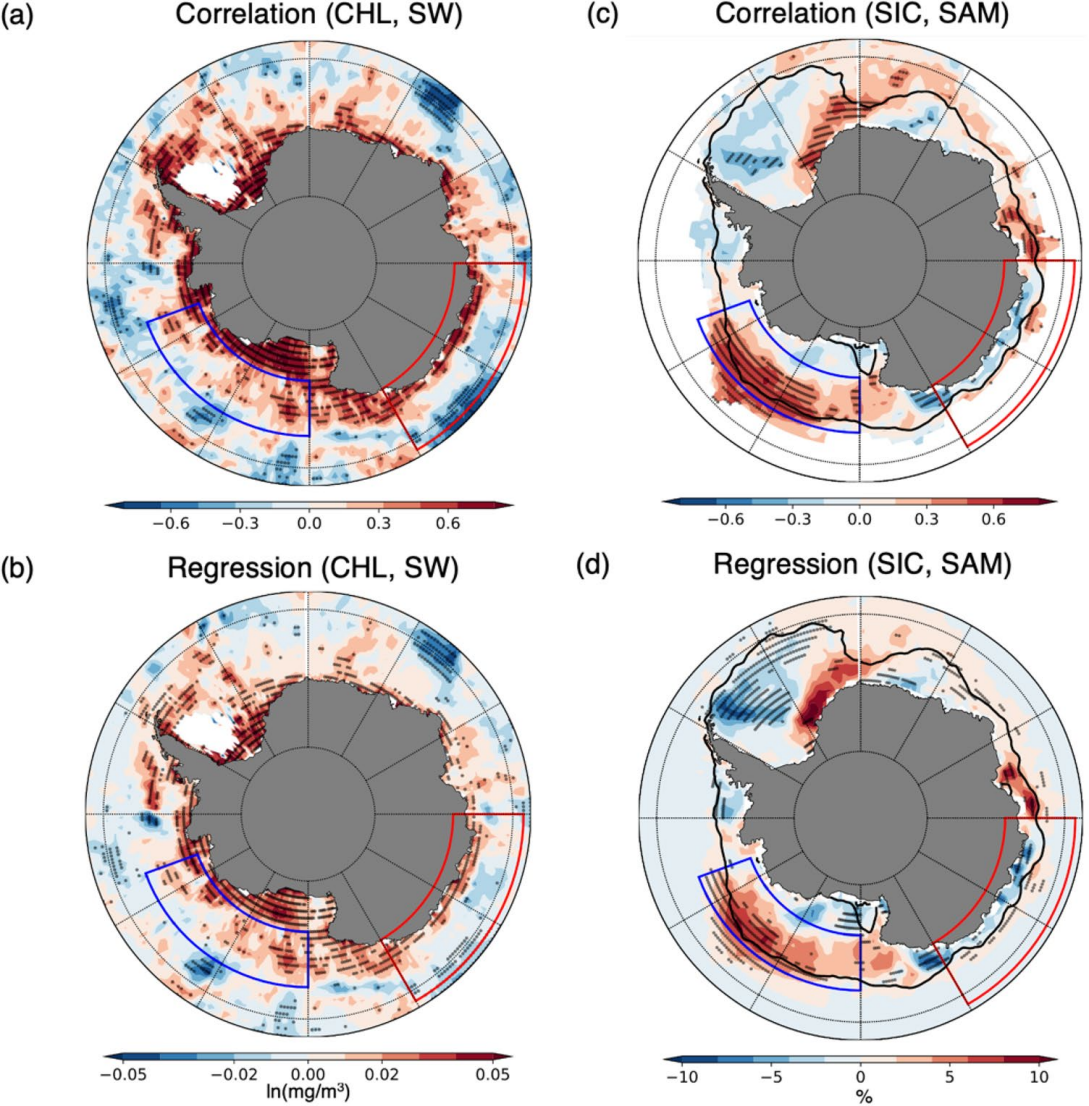
Correlation and regression maps (**a**, **b**) between the chlorophyll concentration and net shortwave flux on the ocean surface and (**c**, **d**) between the SAM index and sea ice concentration. The thick solid line, which is the 15% sea ice concentration line, represents the climatological mean sea ice extent and the 95% significant correlation (*p* value < 0.05) regions are denoted by black dots. White areas indicate the region where satellites have not observed chlorophyll for at least three years due to sea-ice cover or cloud.

To understand these asymmetric biological responses to SAM, identifying the limiting factors is important for the biological growth in each region. Phytoplankton growth is determined by co-limiting factors, such as light and nutrients including nitrate, phosphate, and iron that phytoplankton can grow properly when light and nutrients are sufficiently supplied simultaneously^38^. If phytoplankton growth depends on the light in certain regions, where nutrient supplies are sufficient, the region belonging to this condition can be termed a light-limited region. Similarly, we regard the nutrient-limited region where the chlorophyll changes strongly depend on the nutrient supplies. Therefore, we defined the light-limited regions where the chlorophyll concentration sensitively increased with increased light availability. In Fig. 2a,b the light-limited regions are represented by the positive correlation between the chlorophyll concentration and surface shortwave flux. The sea-ice-extent variability in the Amundsen–Ross Sea region is highest in the Southern Ocean that additional sea-ice melting at spring–summer season could supply the bioavailable iron to the ocean surface, for which the biological response on this region is sensitive to light availability^39, 40^. Consequently, phytoplankton in these areas are more likely to grow well when the light is provided by reduced sea-ice or cloud, and the western Amundsen–Ross Sea region could be regarded as light-limited region (Fig. 2a, b).

The mechanism for the negative correlation between the SAM index and chlorophyll concentration in the Amundsen–Ross Sea is related to the asymmetric sea-ice variability in the Southern Ocean. Sea ice increases in the Amundsen–Ross Sea region during the positive phase of the SAM, particularly near the edge of the sea ice extent (Fig. 2c, d). This sea ice increase is caused by two different mechanisms. First, intensified and poleward-shifted westerly winds at the positive phase of the SAM induce the equatorward Ekman transport, wherein the northward cold and ice advection are enhanced in the Amundsen–Ross Sea region. Second, the Amundsen Sea Low (ASL), which is the dominant stationary low-pressure system in the Southern Ocean, deepens; hence, the cyclonic wind becomes strong and pushes the sea ice within the sea ice extent outside it^10, 41^. The cold advection caused by the westerly change and sea ice drift due to the ASL change and Ekman transport is combined to increase the sea ice in the Amundsen–Ross Sea region. The increased sea ice blocks the shortwave flux; therefore, the reduced shortwave anomalously decreases the chlorophyll concentration because this region is a light-limited region (Fig. 2a, b), which is consistent with the result of Park et al.^42^.

**Figure 3:**
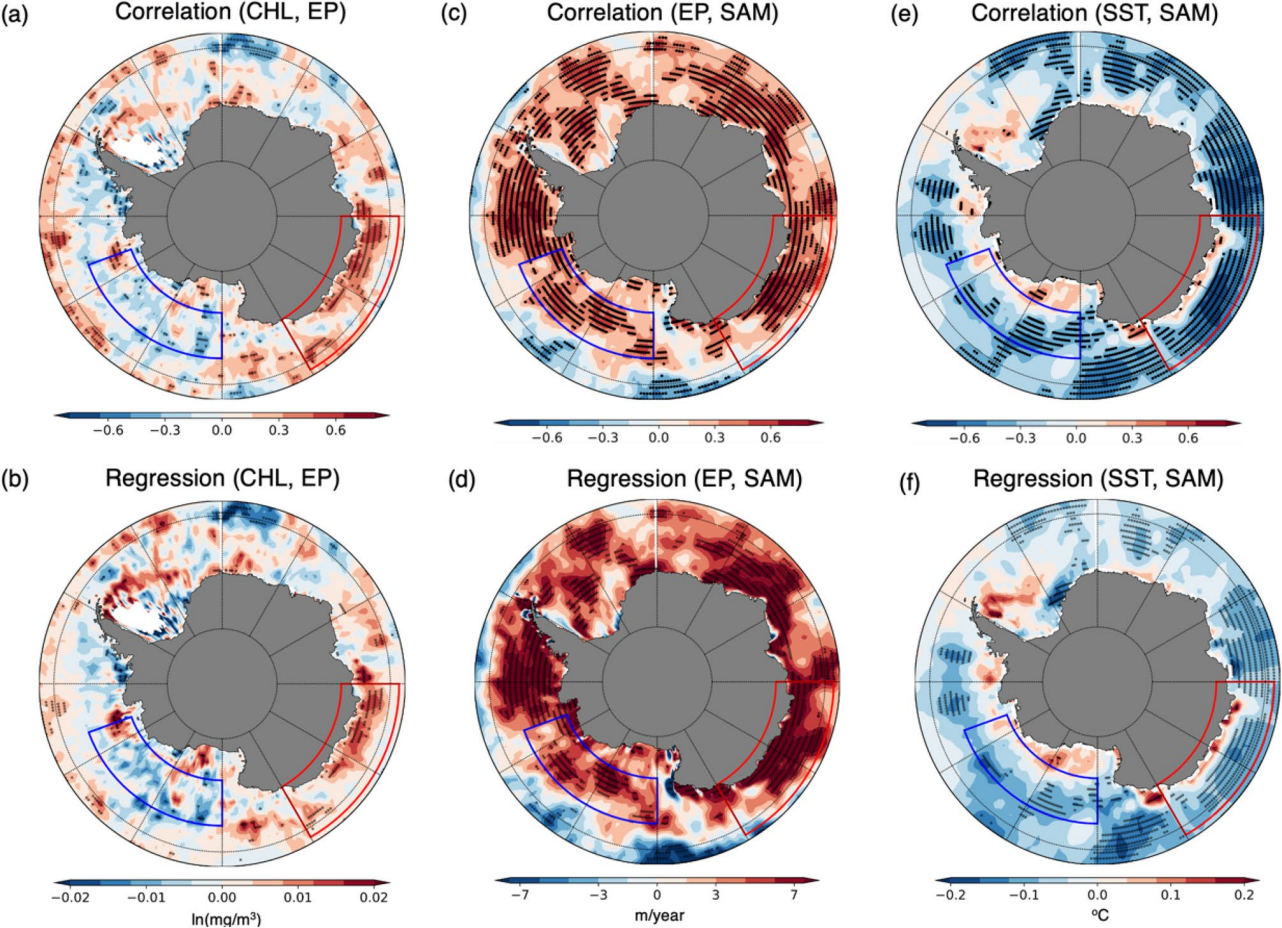
Correlation and regression maps (**a**, **b**) between the chlorophyll concentration and Ekman pumping velocity and between the SAM index and (**c**, **d**) Ekman pumping velocity, (e, f) SST. The 95% significant correlation (*p *value < 0.05) regions are denoted by black dots. White areas indicate the region where satellites have not observed chlorophyll for at least three years due to sea-ice cover or cloud.

Nutrient, especially the iron in the near-surface ocean is usually supplied by Ekman pumping, deep winter mixing, sediment mobilization, rivers, sea-ice, and atmospheric deposition^43^. The nutrient-limited region in the marginal sea of the Southern Ocean could be considered by the positive correlation region between chlorophyll concentration and Ekman pumping velocity. Divergence by Ekman transport generates Ekman pumping, and it could support defining the nutrient-limited region as an indirect method for estimating the amount of surface nutrient supply from the subsurface layer. In particular, the surface nutrient is sensitive to the nutrient supply by the Ekman pumping in the D’Urvill Sea region (Fig. 3a, b). The positive correlation between the chlorophyll anomaly and SAM at the nutrient-limited D’Urville Sea is associated with the intensified and poleward-shifted westerlies at the positive phase of the SAM. Ekman pumping is enhanced by the westerly changes around the Southern Ocean south of the 60° S latitude possibly independent of the longitude (Fig. 3c, d). However, the SST decreases both in the eastern and western parts of the Southern Ocean, with the positive phase of SAM (Fig. 3e, f), implying that colder, nutrient-rich water is supplied to the eastern D’Urville Sea region by the enhanced coastal upwellings. Hence, additional surface nutrient supplies induce the increase in chlorophyll concentration in the D’Urville Sea region only because this region is nutrient-limited (Fig. 3a, b). In the Southern Ocean, dissolved iron is generally insufficient among other nutrients^44, 45^. Therefore, the increased iron supply on the surface by upwelling could be responsible for this chlorophyll change.

**Figure 4:**
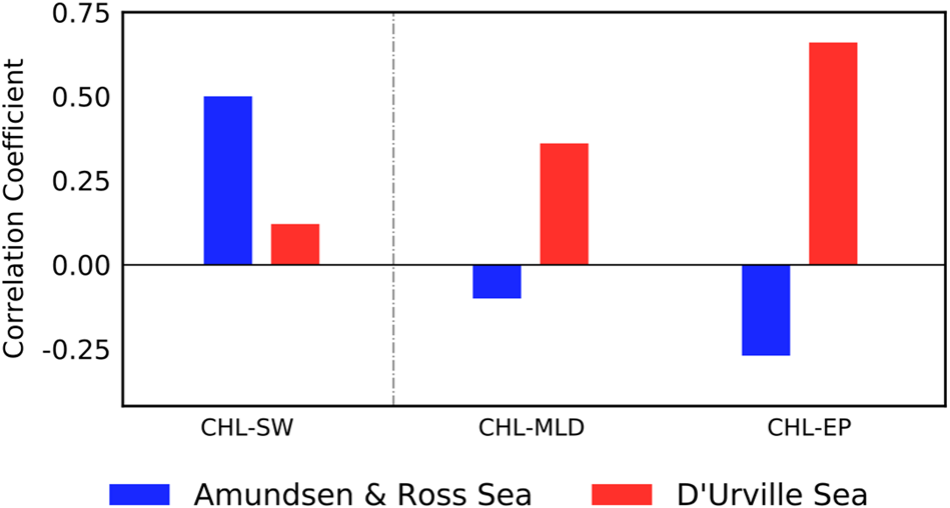
Local correlation coefficients of chlorophyll concentration and net shortwave flux on the ocean surface, MLD, and Ekman pumping velocity. These correlation coefficients are calculated using the spatially averaged time series of each region. The blue and red bars represent the Amundsen-Ross and D’Urville Sea regions, respectively.

The nutrient amount is commonly estimated based on the MLD, which corresponds to the mixing intensity of the water column^14, 27^. In the mixed layer, the cold and nutrient-rich water is usually pulled up to the surface through entrainment. In the marginal sea of the Southern Ocean, we found that MLD exhibits weak relation with chlorophyll changes in austral summer. By comparing the correlations between chlorophyll and MLD and between chlorophyll and Ekman pumping velocity, Ekman pumping velocity is more appropriate for distinguishing this nutrient-limited region than MLD (Fig. 4) to analyze the SAM-driven chlorophyll variations. The climatological mean MLD in the Southern Ocean is deeper than any other oceans due to cold conditions at high latitude and strong winds; hence, the surface nutrient supply is highly controlled by upwelling rather than MLD entrainment.

Two different regions, namely, western and eastern parts of the Southern Ocean, are clearly categorized to light- and nutrient-limited regions, respectively, which is represented as the positive correlation between the chlorophyll–shortwave flux and chlorophyll–Ekman pumping velocity. Moreover, each correlation value is statistically significant with 0.50 and 0.66 values respectively, while each correlation between the chlorophyll–Ekman pumping velocity in the Amundsen-Ross Sea and chlorophyll–shortwave flux in the D’Urville Sea is negative with insignificant small values (Fig. 4). This contrast of two different correlations sign for each region implicated that two different limitation factors, light, and nutrient, are not applied simultaneously for each region.

## Conclusions and discussions

This study has demonstrated the significant relationship between climate mode, SAM, and biological response, phytoplankton changes over the Southern Ocean. Satellite observation and various reanalysis data in the recent two decades that focus on the austral summer (DJF) interannual variability were used herein. Although the SAM’s effect is almost zonally symmetric, the oceanic and biological components respond asymmetrically, and the different chlorophyll responses to the SAM depend on various limitations per region. At the light-limited western Amundsen–Ross Sea region, phytoplankton decreases during the positive phase of SAM. This result is related to the reduced net surface shortwave flux due to sea ice increase. Meanwhile, phytoplankton increase in the nutrient-limited eastern D’Urville Sea region, by the enriched iron flux on the surface owing to the intensified Ekman pumping. These two different mechanisms result in asymmetric chlorophyll response to the SAM.

Lovenduski and Gruber^14^ reported the almost zonally symmetric response of chlorophyll concentration, which is different from the conclusion of the present study, possibly because of the seasonal characteristics and data analysis period. In their study, they used the 8 day daily chlorophyll-*a* data from 1997 to 2004. Herein, we utilized the monthly chlorophyll-*a* concentration data extended to 2019 focusing on interannual variability in the austral summer when it shows the zonal asymmetric response strongly. We reproduced the relationship between SAM and chlorophyll anomaly and found that the seasonality mainly causes different results (Fig. S1). For example, when we calculate the relationship between SAM and chlorophyll using the annual mean data, the zonally asymmetric relation does not appear on the marginal sea. Because of the environmental property change owing to the large sea ice extent retreat in the Amundsen–Ross Sea area during summer, the SAM-driven sea ice increase on the Amundsen–Ross Sea is distinctively shown in the austral summer season, during which the zonal asymmetric response is clearly resolved by the sea ice-driven chlorophyll variation on the Southern Ocean. Our results suggest that the assumption of not changing correlation in time is not valid and the relationship between SAM and chlorophyll will be dependent on the seasonality.

Recent research reported that phytoplankton is expected to increase with the positive trend of SAM over the Southern Ocean^8, 46^ due to the anthropogenic CO_2_ emission. With the positive trend of SAM, the carbon uptake in the Southern Ocean is assumed to increase since the beginning of the twenty-first century^47^ and is predicted to increase in the near future. However, the carbon sink in the Amundsen–Ross Sea region would be mitigated with the sea ice-driven chlorophyll decrease, which coincides with the positive sea ice trend in this region^48^. This reduced chlorophyll will cool the ocean surface through bio-optical feedbacks^49^, and the cold ocean produces better conditions for sea ice formation, consequently reducing chlorophyll once again. Therefore, the biological carbon uptake by the phytoplankton would be decreased in the Amundsen-Ross Sea, and the uptake role of the Southern Oceans would weaken under the positive trend of SAM. Such feedback processes should be taken an account for future projection.

These zonal asymmetric influences of SAM from the sea-level pressure, mixed layer to chlorophyll, are eliciting increasing attention these days. However, they have failed to achieve these asymmetric variabilities at the climate models^27^. Earth system models have not only failed to reproduce the climatological mean state of chlorophyll but also the asymmetric variability of the SAM in the Southern Ocean. This result implies that the present models could not represent accurately the relation between the atmosphere and ocean, probably causing the poor performance of reproducing chlorophyll variability. Meanwhile, the bad performance of implementing the physical components, such as shallow MLD and warm sea surface temperature of the fully coupled model^50^, in the Southern Ocean could be the other specific reason for the bad variability performance of the model. Our analysis results for the asymmetric response indicate the direction of the improvement of the earth system model.

Recently, the biophysical feedback of phytoplankton owing to their variability in the Arctic region has been investigated^24, 25^ and their impacts on the SST are much higher in the Southern Ocean^49^. The warming impacts by biophysical feedbacks would be overestimated in the ESMs if the SAM-driven chlorophyll asymmetric responses are not considered in the models. However, further studies on these biophysical feedbacks at the Southern Ocean should be conducted in the future, and these interactions should be integrated into the earth system models to reproduce better mean state and variabilities of the physical and biogeochemical components on the models. Also, the recent studies about sea-ice related biogeochemical impacts^51–53^ have been conducted that the interactions between sea-ice changes and plankton phenology such as photosynthesis at low light condition and grazing need to be cooperated with the ESMs to represent the precise limitation of phytoplankton growth. Therefore, the asymmetric biological impact on climate variability improves the understanding of phytoplankton with limitation factors and is indicative of the future direction of the Earth System Model.

## Supplementary Information

Supplementary Information 1.

## Data Availability

All figures were generated by using software package Python with the matplotlib and basemap modules (https://matplotlib.org/ , https://matplotlib.org/basemap/). The map coastlines are derived by the Global Self-consistent, Hierarchical, High-resolution Geography (GSHHG) Database (https://www.soest.hawaii.edu/pwessel/gshhg/), which has been distributed under the GNU Lesser General Public License, and is provided with the basemap Python module.
