# Supplementary material for: Zonally asymmetric phytoplankton response to the Southern annular mode in the marginal sea of the Southern ocean
Source: Sci Rep. 2021 May 13;11:10266. doi: 10.1038/s41598-021-89720-4 (PMC8119945; doi:10.1038/s41598-021-89720-4)
Supplement: Supplementary file 1 — Supplementary Information 1. [file 41598_2021_89720_MOESM1_ESM.pdf]

Supporting Information for

Zonally asymmetric phytoplankton response to the Southern Annular Mode in the marginal sea of the Southern Ocean

Authors : Kyung Min Noh[1], Hyung-Gyu Lim[1,2], and Jong-Seong Kug[1]

[1]Division of Environmental Science and Engineering, Pohang University of Science and Technology (POSTECH), 77 Cheongam-Ro Nam-Gu, Pohang 790-784, South Korea

[2]Princeton University/Atmospheric and Oceanic Sciences Program, Princeton, NJ 08540, USA

List of Figures:

Figure S1: Correlation and regression maps between SAM index and chlorophyll anomalies in each season

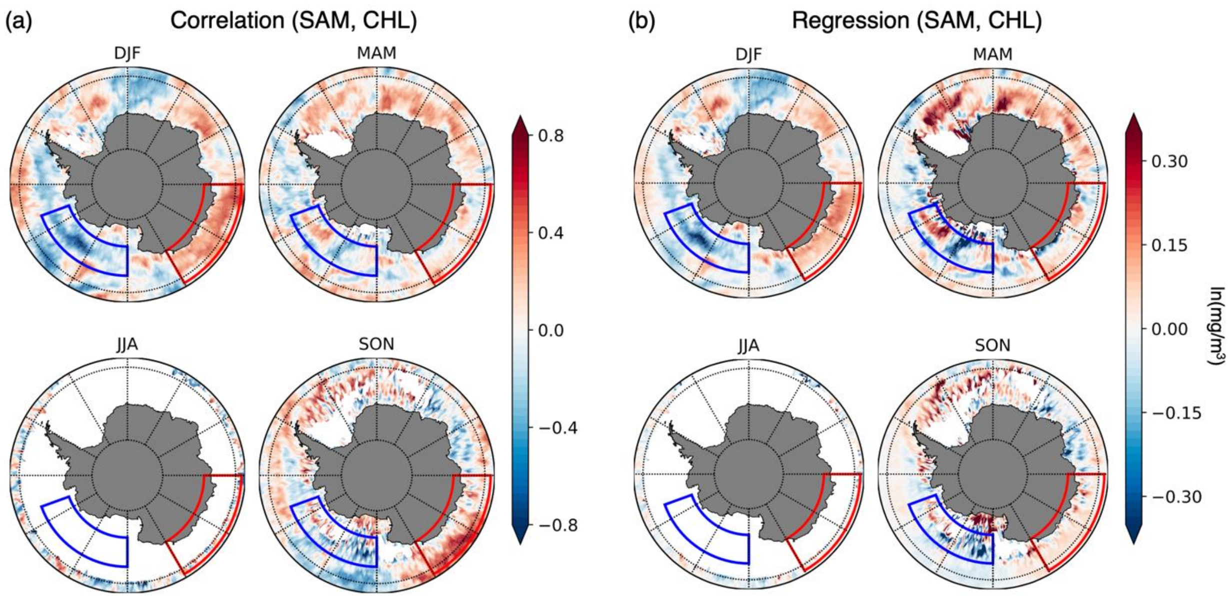

Figure S1 Correlation (a) and regression (b) maps between the SAM and chlorophyll concentration anomaly in each season. The western and eastern parts of the Southern Ocean are shown using blue and red boxes, respectively.
